# Comprehensive analysis of the prognostic, immunological, and diagnostic roles of SIRT1 in pan-cancer and its validation in KIRC

**DOI:** 10.3389/fimmu.2024.1501867

**Published:** 2025-01-08

**Authors:** Qi Liu, Songxian Sun, Chunxiang Zhou, Houxi Xu

**Affiliations:** ^1^ School of Chinese Medicine, Nanjing University of Chinese Medicine, Nanjing, China; ^2^ Key Laboratory of Acupuncture and Medicine Research of Ministry of Education, Nanjing University of Chinese Medicine, Nanjing, China

**Keywords:** SIRT1, pan-cancer, immunity, prognosis, biomarkers

## Abstract

**Background:**

Disturbances in DNA damage repair may lead to cancer. SIRT1, an NAD+-dependent deacetylase, plays a crucial role in maintaining cellular homeostasis through the regulation of processes such as histone posttranslational modifications, DNA repair, and cellular metabolism. However, a comprehensive exploration of SIRT1’s involvement in pan-cancer remains lacking. Our study aimed to analyze the role of SIRT1 in pan-cancer to gain a more comprehensive understanding of its role in multiple malignancies.

**Methods:**

We systematically examined the role of SIRT1 in pan-cancer by analyzing data from The Cancer Genome Atlas (TCGA) and Genotype-Tissue Expression (GTEx) databases. Various tools, including R, Cytoscape, HPA, Archs4, TISIDB, cBioPortal, STRING, GSCALite, and CancerSEA, were used to integrate and analyze SIRT1 gene expression, prognosis, protein interactions, signaling pathways, immune infiltration, and other relevant information. Furthermore, we validated the differential expression of SIRT1 in normal human kidney cells and kidney cancer cell lines via experimental verification.

**Results:**

SIRT1 expression was significantly reduced in various cancers and was different across molecular and immune subtypes. SIRT1 is intricately linked to numerous cancer pathways. In most cancer types, increased SIRT1 expression is positively associated with eosinophils, helper T cells, central memory T cells, effector memory T cells, γδ T cells, and Th2 cells. SIRT1 expression is significantly correlated with immune regulatory factors across various cancer types. Quantitative reverse transcription polymerase chain reaction (qRT–PCR) and Western blot (WB) analyses confirmed that SIRT1 is differentially expressed in kidney renal clear cell carcinoma (KIRC).

**Conclusions:**

Using an integrative approach involving bioinformatics analysis and experimental validation, we clarified the potential roles and mechanisms of SIRT1 in pan-cancer, providing a theoretical basis for the development of SIRT1-targeted therapies in clinical applications.

## Introduction

DNA damage can result from a variety of environmental factors, including ultraviolet radiation, radioactive substances, chemicals (such as benzene and asbestos), and lifestyle choices (such as smoking and dietary habits) (1). These factors can alter DNA structure and function through distinct molecular mechanisms, ultimately leading to genetic mutations or chromosomal abnormalities. Ultraviolet radiation and radioactive substances can directly harm the DNA double helix, causing base pair mutations or double-strand breaks. Chemical substances can indirectly induce DNA damage by forming DNA adducts or triggering oxidative stress. Accumulated DNA damage can cause permanent genetic alterations and increase the risk of cancer (2).

In eukaryotic cells, the DNA damage response (DDR) is a complex regulatory system that evolved to maintain genetic integrity and prevent the accumulation of damaged DNA (3). This mechanism evolved in response to environmental stressors such as ultraviolet rays, radioactive radiation, chemicals, and free radicals to support life and ensure reproduction. Eukaryotic cells have developed efficient monitoring and repair mechanisms to ensure genome stability. The core goal of the DDR mechanism is to accurately identify DNA damage and promptly initiate the repair process (4). The process starts with the detection of DNA damage by a complex formed by recognition proteins such as RAD9, RAD1, and HUS1, which act as ‘sentinels’ to sense damage and activate downstream kinase pathways (5). ATM and ATR kinases are rapidly recruited to the damage site upon detection of DNA damage, transmitting repair signals by phosphorylating downstream effector molecules, such as CHK1, CHK2, p53, and BRCA1 (6, 7). This phosphorylation cascade triggers multiple signaling pathways, halting the cell cycle to allow time for DNA repair. Additionally, the DDR mechanism involves the regulation of chromatin structure (8). Following DNA damage, the chromatin environment is restructured to facilitate access for repair proteins to the damaged site. ATM and ATR can phosphorylate histone H2AX to mark damaged areas and recruit additional repair proteins to this site. SIRT1 plays a pivotal role throughout the DDR process.

SIRT1, a member of the sirtuin protein family, is located in the 7q31.3 region of human chromosomes. It is involved in various cellular physiological processes through its deacetylase activity, including chromatin remodeling, DNA repair, the regulation of gene expression, cell metabolism, and aging (9–11). Recent studies have demonstrated that SIRT1 plays diverse roles in tumorigenesis and cancer progression. For example, Jin et al. reported that increased SIRT1 expression was linked to poorer prognosis in breast cancer, whereas Zhang et al. reported a significant association between high SIRT1 expression and reduced survival in esophageal squamous cell carcinoma (12, 13). These findings highlight the complex role of SIRT1 as both an oncogene and a tumor suppressor, depending on the cancer context (14–17).

SIRT1 regulates gene transcription and maintains chromosomal stability by deacetylating histone and nonhistone substrates. It plays a crucial role in cell signaling pathways, particularly in response to DNA damage and oxidative stress (18). In the DNA damage response mechanism, SIRT1 influences cell cycle progression and the response to damage by deacetylating specific transcription factors and repairing proteins such as p53 (19). The activity and expression of SIRT1 are central to maintaining the cellular metabolic balance and responding to environmental stress (20). Loss of SIRT1 activity in animal models leads to metabolic disorders and accelerated aging, whereas excessive activation is linked to extended lifespan and antiaging effects (21). Therefore, SIRT1 is a key player in maintaining chromosomal stability and cellular function and is a significant target for research on aging and related diseases, such as cancer, neurodegenerative diseases, and metabolic disorders (22). Recent studies have demonstrated a strong correlation between abnormal SIRT1 expression and dysfunction and the development and prognosis of various malignant cancers (14, 15). For example, Wang et al. reported that elevated levels of SIRT1 in solid tumors, such as liver and lung cancers, were linked to poorer overall survival rates (16). Uzelac et al. revealed an inverse relationship between SIRT1 expression and overall survival, progression-free survival, TNM stage, and lymph node metastasis in breast cancer (17). Similarly, increased SIRT1 expression has been associated with an unfavorable prognosis in colorectal cancer, esophageal squamous cell carcinoma, and gastric cancer (13, 23, 24). While the role of SIRT1 in specific cancer types has been extensively researched, there is a lack of comprehensive studies on its involvement in pan-cancers. Therefore, investigating the mechanisms of SIRT1 in pan-cancer is crucial for advancing cancer treatments.

In this study, we conducted a comprehensive analysis via multiple databases, including TCGA, GTEx, TISIDB, cBioPortal, STRING, GSCALite, and CancerSEA, to explore the gene expression, prognosis, protein interactions, and associated signaling pathways of SIRT1 in pan-cancer. Furthermore, we explored the correlation between SIRT1 expression and immune cell infiltration across 33 different types of cancer. Finally, we validated SIRT1 expression in cancer cell lines through qRT–PCR and WB experiments. Our research highlights the crucial role of SIRT1 in pan-cancer studies and establishes a foundation for uncovering its potential involvement in cancer development and therapeutic strategies.

## Materials and methods

### Expression of SIRT1 in pan-cancer

The Human Protein Atlas (HPA; https://www.proteinatlas.org/) offers information on human protein expression and localization in different tissues and cells, with the goal of advancing proteomic research (25). The HPA database was used to gather data on SIRT1 mRNA and protein expression in human tissues. The Harmonizome database (https://maayanlab.cloud/Harmonizome/) integrates diverse biomedical datasets to provide gene and protein expression details under various biological conditions, aiding in the identification of biomarkers and therapeutic targets. The Harmonizome database was used to collect data on SIRT1 mRNA expression in various tissues and cell lines (26). The Tumor Genome Atlas (TCGA, https://cancergenome.nih.gov) compiles sequencing data from numerous human cancer samples to support comprehensive genomic profiling of cancers with the aim of enhancing cancer diagnosis, treatment, and prevention (27). Genotype-Tissue Expression (GTEx, https://gtexportal.org/) is a public service platform that facilitates research on gene expression and genetic regulation and provides gene expression data across different human tissues (28). SIRT1 mRNA expression was analyzed in 33 cancer types via data from the TCGA and GTEx databases. These cancer types include adrenocortical carcinoma (ACC), bladder urothelial carcinoma (BLCA), breast invasive cancer (BRCA), cervical squamous cell carcinoma and cervical adenocarcinoma (CESC), cholangiocarcinoma (CHOL), colon adenocarcinoma (COAD), diffuse large B-cell lymphoma (DLBC), esophageal cancer (ESCA), glioblastoma multiforme (GBM), head and neck squamous cell carcinoma (HNSC), renal pheochromocytoma (KICH), renal hyaline renal cell carcinoma (KIRC), renal papillary cell carcinoma (KIRP), acute myeloid leukemia (LAML), low-grade glioma (LGG), hepatocellular carcinoma (LIHC), lung adenocarcinoma (LUAD), lung squamous cell carcinoma (LUSC), mesothelioma (MESO), ovarian serous cystadenocarcinoma (OV), pancreatic adenocarcinoma (PAAD), pheochromocytoma and paraganglioma (PCPG), prostate adenocarcinoma (PRAD), rectal adenocarcinoma (READ), sarcoma (SARC), cutaneous melanoma (SKCM), gastric adenocarcinoma (STAD), testicular germ cell tumor (TGCT), thyroid cancer (THCA), thymoma (THYM), uterine corpus endometrium carcinoma (UCEC), uterine sarcoma (UCS), and uveal melanoma (UVM).

### Receiver operating characteristic curve analysis of SIRT1 in pan-cancer

The ROC curve is a vital tool for assessing the performance of a classification model by illustrating the relationship between the true positive and false positive rates at various threshold settings to showcase the diagnostic efficiency of the model (29). In this study, receiver operating characteristic (ROC) curves were used to assess the diagnostic efficacy of SIRT1 in 33 cancer types. The pROC package (v1.18.5) in R was used to generate ROC curves, whereas the ggplot2 package was used for visualization (30). Furthermore, the area under the curve (AUC) of the ROC curve was calculated as a measure of diagnostic performance. The AUC value ranged from 0 to 1, with higher values indicating better diagnostic performance. AUC values between 0.5 and 0.7 suggest low accuracy, values between 0.7 and 0.9 indicate high accuracy, and values greater than 0.9 represent models with extremely high diagnostic accuracy.

### Survival analysis of SIRT1 in pan-cancer

Kaplan–Meier (KM) survival analysis is a commonly employed statistical approach for comparing survival rates across different groups. In this study, the survival package in R software was used to conduct KM survival analysis on the high- and low-expression groups of SIRT1 in 33 cancer types, including overall survival (OS), disease-specific survival (DSS), and progression-free interval (PFI) (31). The Cox regression model was used to determine *p*-values and evaluate the significance of survival disparities. By integrating the Survminer and ggplot2 packages, hazard ratios (HR), 95% confidence intervals, and *p*-values were computed and visually represented (32).

### Associations between SIRT1 expression and immune subtypes in pan-cancer

The TISIDB database serves as a comprehensive platform for analyzing interactions between cancer and the immune system, consolidating various data types from public databases, such as gene expression, immune subtypes, and immunotherapy markers (33). Through the integration of these datasets, TISIDB offers a user-friendly interface for investigating the expression and clinical relevance of specific genes across diverse cancer immune subtypes. This study utilized the ‘subtype’ module of TISIDB to explore the correlation between SIRT1 gene expression and the molecular and immune subtypes of 33 cancers. Specifically, the expression of SIRT1 mRNA in six immune subtypes (C1: wound healing type, C2: IFN-γ dominant type, C3: inflammatory type, C4: lymphocyte depletion type, C5: immune quiet type, C6: TGF- β-dominant type) was analyzed to reveal its behavioral patterns in different immune environments.

### Variation analysis of SIRT1 in pan-cancer

cBioPortal is a public online resource that is specifically used to query cancer genomics datasets, providing a wide range of cancer genome information, including information on gene mutations, copy number variations, and expression differences (34). This database combines data from multiple cancer genome research projects around the world, such as the TCGA, and supports a comprehensive analysis across cancer types. cBioPortal provides an interactive interface and versatile modules that enable cancer researchers to explore and analyze complex genomic data intuitively. The variation in the SIRT1 gene in various cancers, including the frequency and mutation sites of its somatic mutations, was explored via the cBioPortal website to reveal the genomic variation characteristics of the SIRT1 gene in different cancer types.

### Protein–protein interaction network analysis of SIRT1

The STRING database is a comprehensive resource for protein–protein interaction (PPI) networks containing both known and predicted interaction information (35). It covers a wide range of species and assigns a confidence score to each interaction, aiding researchers in evaluating the reliability of the data. STRING’s user-friendly interface supports various analyses, such as network views, functional enrichment analysis, and genome context tools. In this study, protein interaction data associated with SIRT1 were gathered from the STRING database to construct a PPI network. The confidence threshold was set to 0.7 to filter significant interaction data for network analysis. The network data were subsequently imported into Cytoscape (v3.10.1) for visualization and analysis (36). Key network modules were identified via Cytoscape’s cytoHubba plug-in, and the top 10 hub genes ranked via the MCC method were highlighted (37).

### Functional enrichment analysis of SIRT1

ClusterProfiler is an R software package specifically developed for statistical methods and visualizing tools for comparative clustering and enrichment analysis of biological terms (38). It supports various biological annotation resources, such as Gene Ontology (GO) and Kyoto Encyclopedia of Genes and Genomes (KEGG) pathways, making it a robust tool for studying the functional characteristics and pathway associations of gene sets. In this study, we utilized the clusterProfiler package to conduct GO function and KEGG pathway enrichment analyses of genes closely linked to the SIRT1 gene. The ggplot2 package was subsequently employed to visually represent the analysis results via bubble charts.

### Gene set enrichment analysis of SIRT1

Gene set enrichment analysis (GSEA) is a computational method used to assess whether a predefined set of genes, such as a biological pathway, is significantly enriched in gene expression data. GSEA helps to identify the overall impact of gene expression changes on biological functions, revealing the activation or inhibition of biological processes. In this study, the clusterProfiler package was used to perform GSEA on SIRT1 to compare the differences in biological pathways between the SIRT1 high and low expression groups. The significance criterion was set at a corrected *P*-value <0.05, and the stability of the analysis was ensured by performing 1000 genome permutations. The top 10 significantly enriched genes were visualized as mountain plots, and the GSEA results were displayed via ggplot2 software.

### Analysis of the functional status of SIRT1 in pan-cancer

CancerSEA is a specialized database created to reveal the distinct functional states of cancer cells at the single-cell level, encompassing processes such as proliferation, migration, and invasion (39). By amalgamating single-cell expression data from diverse cancer types, the database allows researchers to explore the impact of specific genes on cancer cell behavior and function. Moreover, CancerSEA offers tools for quantitatively evaluating the functional diversity of cancer cells, thereby enhancing our understanding of the intricate biology of cancer. Using the CancerSEA database, an analysis of the functional status of SIRT1 in various cancers was conducted to investigate its association with traits such as invasion, metastasis, proliferation, EMT, angiogenesis, and apoptosis across 18 cancer types. Significant correlations between SIRT1 and the functional status of these cancers were identified by applying a correlation strength threshold of 0.3 and a *P*-value <0.05.

### Immunogenomic analysis of SIRT1 in pan-cancer

Gene set variation analysis (GSVA) is an R software package used to assess gene set variation within a sample, facilitating unsupervised extraction and quantification of gene set activity (40). This method offers an alternative to enrichment analysis for interpreting gene expression data, particularly for biological pathway and functional analyses. The GSVA package was used to investigate the relationships between the SIRT1 gene and various factors, including tumor-infiltrating lymphocytes (TILs), immunostimulators, immunoinhibitors, MHC molecules, chemokines, and chemokine receptors, in 33 types of cancer. The significance of these correlations was evaluated through Spearman correlation analysis, with *P* values <0.05 considered statistically significant. The correlation results were visually represented as heatmaps via the ggplot2 software package.

### Cell culture and treatment

The ACHN and HK-2 cell lines were procured from the Cell Bank of the Shanghai Institute of Cell Biology, Chinese Academy of Sciences. ACHN cells were cultured in RPMI-1640 medium supplemented with 10% FBS, whereas HK-2 cells were grown in DMEM supplemented with 10% FBS. Cell transfection was conducted via the Lipofectamine 2000 reagent following the manufacturer’s instructions. The cells were plated in 6-well plates and transfected when they reached 30% confluence. Following transfection, the cells were incubated at 37°C for 48 hours and subsequently subjected to quantitative reverse transcription polymerase chain reaction (qRT–PCR) and other experimental procedures.

### RNA extraction and qRT–PCR

Total RNA was extracted from 1×10^6 cells via TRIzol reagent according to the manufacturer’s protocol. The primers used for qRT–PCR, including those for SIRT1 and GAPDH, were obtained from Shanghai Jierui Bioengineering Co., Ltd. (Generay, Shanghai, China). The forward primer for NAT1 was 5’-TAGACACGCTGGAACAGGTTGC-3’, and the reverse primer was 5’-CTCCTCGTACAGCTTCACAGTC-3’. The forward primer for GAPDH was 5’-GTCTCCTCTGACTTCAACAGCG-3’, and the reverse primer was 5’-ACCACCCTGTTGCTGTAGCCAA-3’. The qRT–PCR cycling conditions were as follows: initial denaturation at 95°C for 6 minutes, followed by 40 cycles of 95°C for 10 seconds for denaturation and 58°C for 30 seconds for annealing. Relative gene expression levels were normalized to an internal control and calculated via the 2^−ΔΔCt^ method.

### Western blot

After the cells were washed with PBS, they were lysed in RIPA buffer for 10 minutes, followed by centrifugation at 4°C to collect the supernatant. The protein concentration in the supernatant was measured via a BCA assay kit. The quantified proteins were then mixed with loading buffer, denatured by heating, and separated by SDS–PAGE, followed by membrane transfer within 120 minutes. The membrane was blocked with 5% nonfat milk for 1 hour and then incubated overnight at 4°C with primary antibodies against SIRT1 (1:1,000) and β-actin (1:5,000). The next day, the membrane was incubated with HRP-conjugated secondary antibodies (1:10,000) at room temperature for 1 hour, and the protein bands were detected via enhanced chemiluminescence (ECL) reagent.

### Statistical analysis

All the statistical analyses were conducted via R software (41). *P*-value <0.05 were considered statistically significant.

## Results

### Expression of SIRT1 in pan-cancer

**Figure 1:**
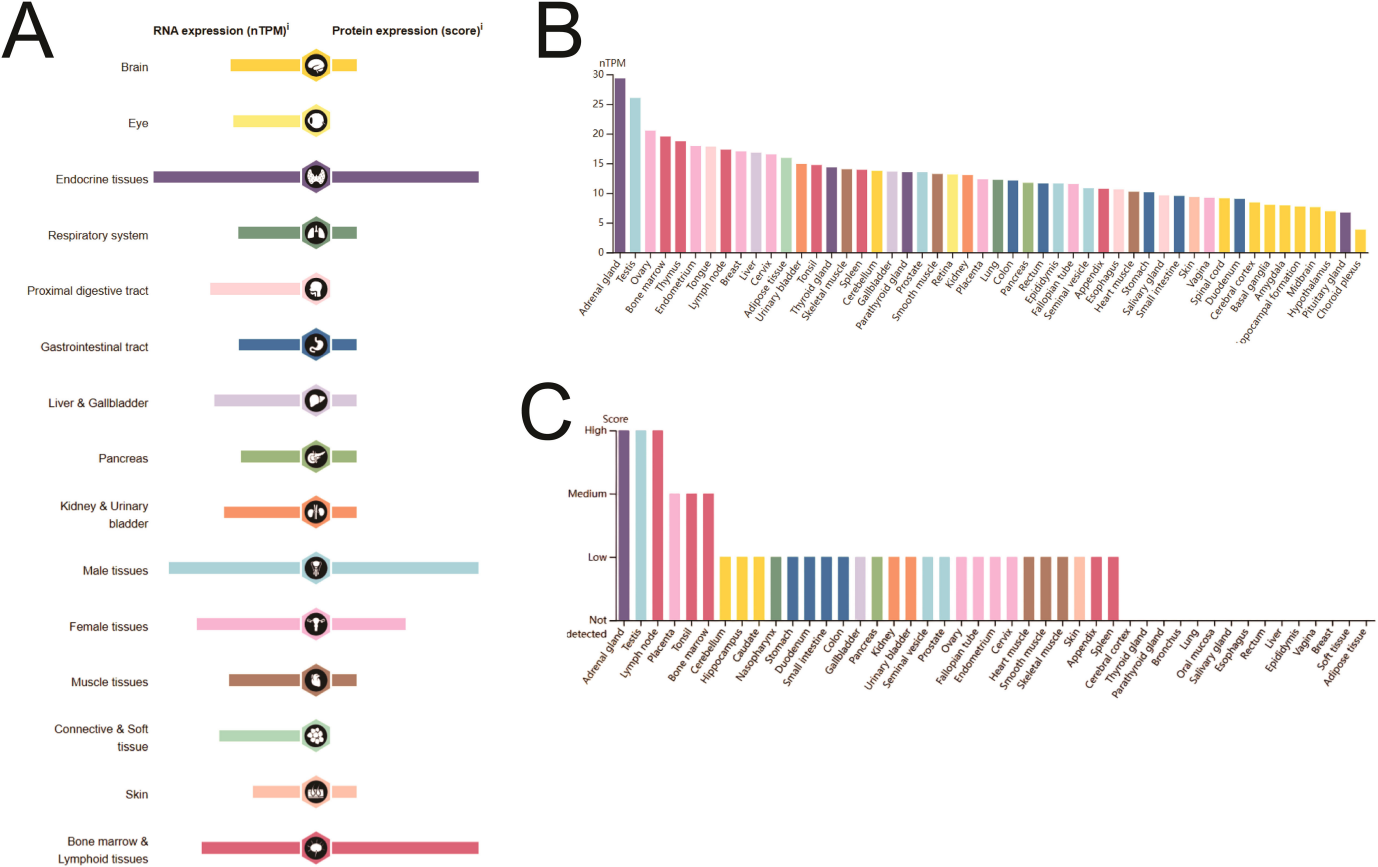
SIRT1 mRNA and protein expression in human organs and tissues. **(A)** Details of SIRT1 mRNA and protein expression in human organs and tissues. **(B)** SIRT1 mRNA expression in human tissues. **(C)** SIRT1 protein expression in human tissues.

SIRT1 is expressed at the mRNA and protein levels in various organs and tissues of the human body (**Figure 1A**). Analysis of the HPA database revealed that SIRT1 mRNA is predominantly expressed in the adrenal gland, testis, ovary, bone marrow, thymus, endometrium, lymph nodes, chest, and liver. In contrast, SIRT1 protein was detected primarily in the adrenal gland, testis, lymph nodes, placenta, tonsils, and bone marrow (**Figures 1B, C**). Additional information regarding SIRT1 mRNA expression in tissues and cell lines is provided in **Supplementary Figure S1**.

**Figure 2:**
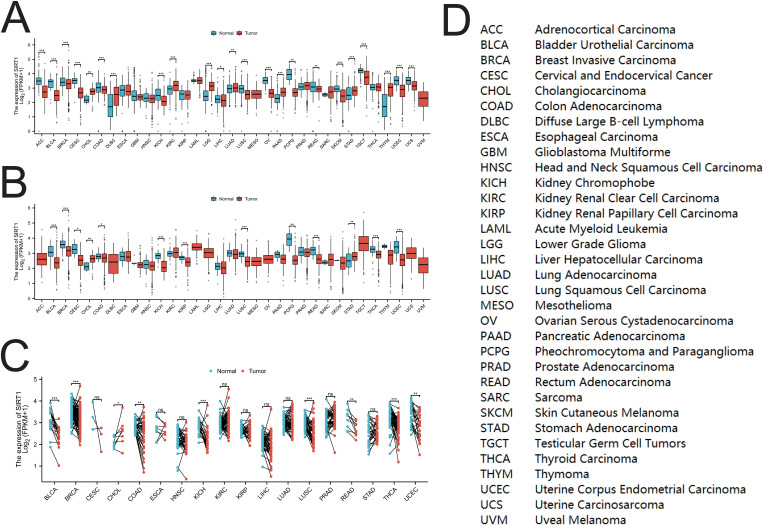
SIRT1 mRNA expression in pan-cancer. **(A)** Expression of SIRT1 between cancer tissues and normal tissues in 33 types of cancer. **(B)** Expression of SIRT1 between cancer tissues and para-cancerous tissues in 33 types of cancer; **(C)** Cancer tissues and paired para-cancerous tissues in 18 types of cancer SIRT1 mRNA expression among tissues. **(D)** Abbreviations of various cancers in TCGA. *p < 0.05, **p < 0.01, ***p < 0.001. ns, not significant.

SIRT1 mRNA expression in multiple cancer types was evaluated via the TCGA and GTEx databases. As shown in **Figure 2A**, compared with those in normal tissues, the mRNA levels of SIRT1 were significantly lower in ACC, BLCA, BRCA, CESC, COAD, KICH, LIHC, LUSC, OV, PCPG, READ, SKCM, TGCT, UCEC, and UCS (P <0.05), whereas they were significantly greater in CHOL, DLBC, KIRC, LGG, LUAD, PAAD, STAD, and THYM (P <0.01). Compared with that in paracancerous tissues (**Figure 2B**), SIRT1 mRNA expression was significantly lower in BLCA, BRCA, CESC, COAD, KICH, KIRP, LUSC, PCPG, READ, THCA, and UCEC tissues (P <0.05), whereas it was significantly greater in CHOL and STAD tissues (P <0.01). Comparison with paired paracancerous tissues (**Figure 2C**) revealed that SIRT1 mRNA levels were increased in CHOL but decreased in BLCA, BRCA, COAD, KICH, LUSC, READ, THCA, and UCEC (P <0.05) (**Figure 2D**). Differential expression of SIRT1 in pan-cancer can be found in **Supplementary Table S1**.

### Diagnostic value of SIRT1 in pan-cancer

**Figure 3:**
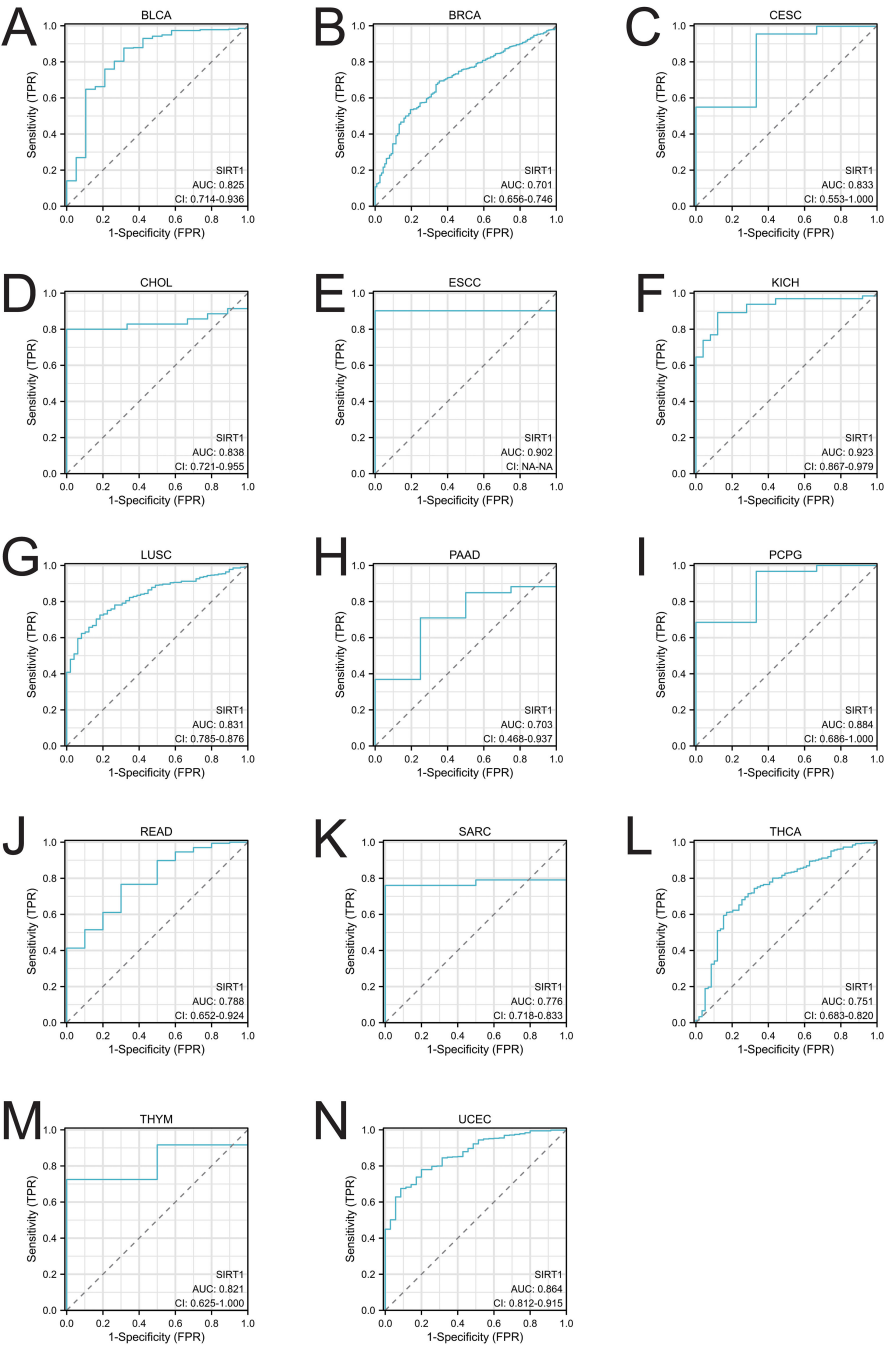
Receiver operating characteristic (ROC) curve of SIRT1 in 14 cancer types. Cancers with an AUC of SIRT1 > 0.7: **(A)** BLCA, **(B)** BRCA, **(C)** CESC, **(D)** CHOL, **(E)** ESCC, **(F)** KICH, **(G)** LUSC, **(H)** PAAD, **(I)** PCPG, **(J)** READ, **(K)** SARC, **(L)** THCA, **(M)** THYM, and **(N)** UCEC.

**Figures 3A-N** demonstrate the diagnostic value of SIRT1 in various cancers. The AUC values of SIRT1 exceeded 0.7 in 14 cancers, especially in KICH and ESCC, where the AUC values reached 0.923 and 0.902, respectively, indicating extremely high diagnostic efficacy. Other cancer types, such as BLCA (AUC = 0.825), BRCA (AUC = 0.701), CESC (AUC = 0.833), CHOL (AUC = 0.838), LUSC (AUC = 0.831), PAAD (AUC = 0.703), PCPG (AUC = 0.884), READ (AUC = 0.788), SARC (AUC = 0.776), THCA (AUC = 0.751), THYM (AUC = 0.821), and UCEC (AUC = 0.8640), also showed good diagnostic ability for SIRT1. The AUC values of SIRT1 in pan-cancer can be found in **Supplementary Table S2**.

### Survival analysis of SIRT1 in pan-cancer

**Figure 4:**
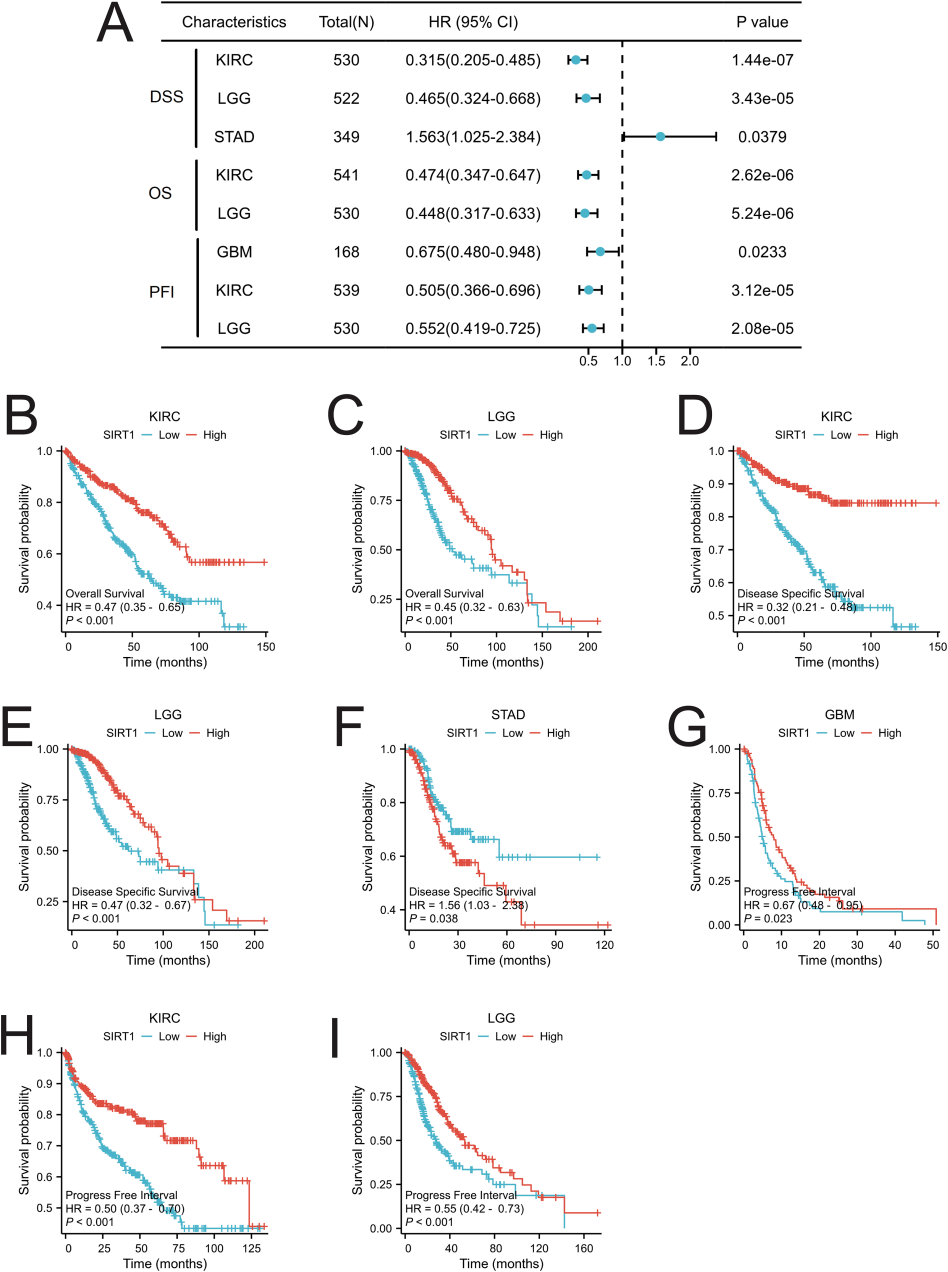
Prognostic analysis of SIRT1 in pan-cancer. **(A)** Prognostic analysis of SIRT1 in pan-cancer, including overall survival (OS), disease-specific survival (DSS), and progression-free interval (PFI); **(B)** OS of SIRT1 in KIRC; **(C)** OS of SIRT1 in LGG; **(D)** DSS of SIRT1 in KIRC; **(E)** DSS of SIRT1 in LGG; **(F)** DSS of SIRT1 in STAD; **(G)** PFI of SIRT1 in GBM; **(H)** PFI of SIRT1 in KIRC; **(I)** PFI of SIRT1 in LGG.

To assess the prognostic significance of SIRT1 across multiple cancer types, we conducted a KM analysis. Cox regression analysis of the 33 different cancer types revealed a significant correlation between SIRT1 expression and OS in KIRC and LGG (**Figure 4A**). Specifically, the high-expression group had notably better OS than did the low-expression group (**Figures 4B, C**). We found that SIRT1 expression was significantly associated with DSS in KIRC, LGG, and STAD (**Figure 4A**). Notably, SIRT1 exhibited a protective role in KIRC and LGG but acted as a risk factor in STAD (**Figures 4D-F**). Moreover, our analysis revealed a significant association between SIRT1 expression and the PFI in KIRC, LGG, and GBM (**Figure 4A**), with SIRT1 showing a protective effect in GBM, KIRC, and LGG (**Figures 4G-I**).

### Expression of SIRT1 in different immune and molecular subtypes

**Figure 5:**
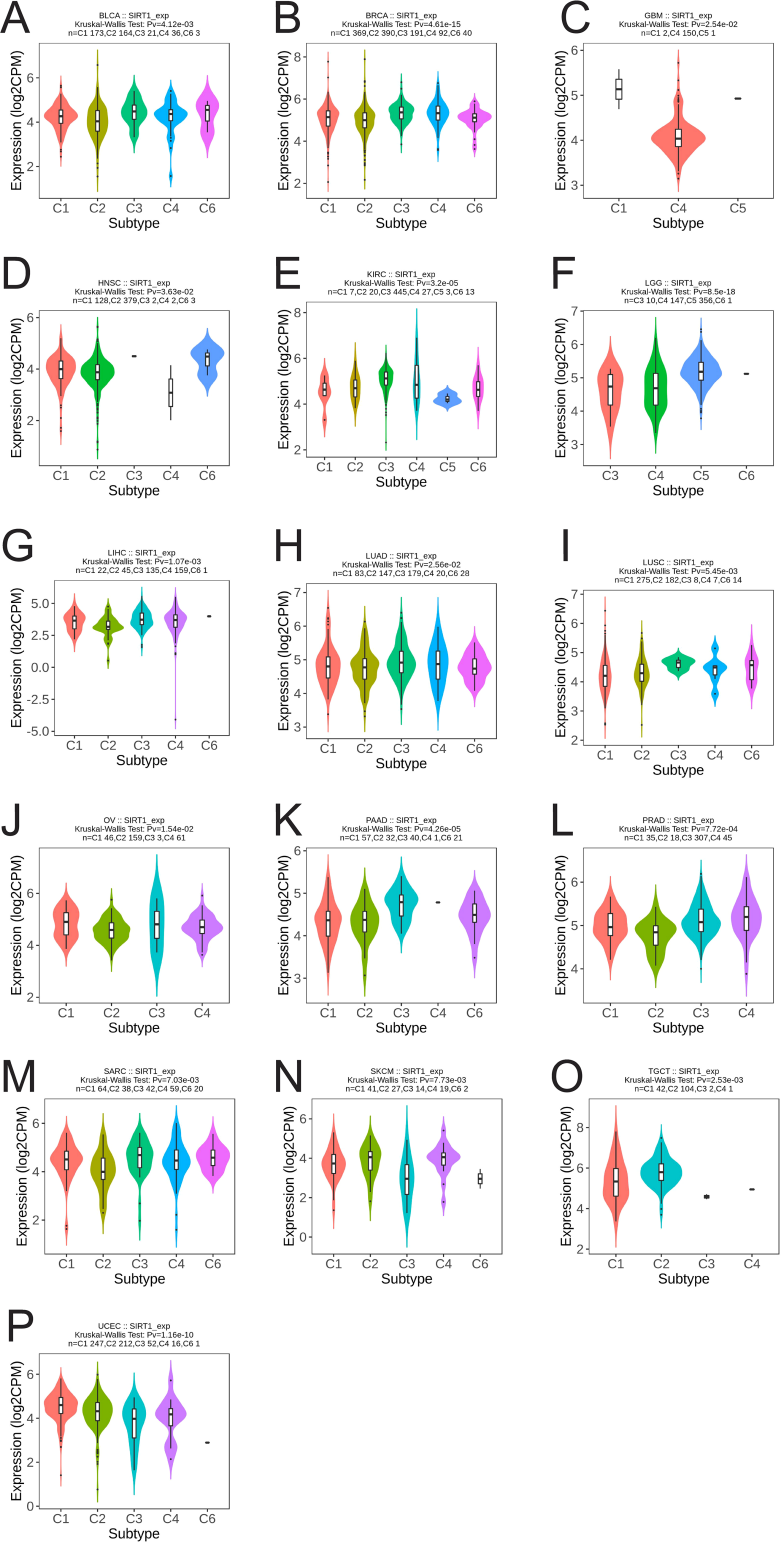
Correlation analysis between SIRT1 expression and immune subtypes in 16 cancers. **(A)** BLCA, **(B)** BRCA, **(C)** GBM, **(D)** HNSC, **(E)** KIRC, **(F)** LGG, **(G)** LIHC, **(H)** LUAD, **(I)** LUSC, **(J)** OV, **(K)** PAAD, **(L)** PRAD, **(M)** SARC, **(N)** SKCM, **(O)** TGCT, **(P)** UCEC. C1 (wound healing), C2 (IFN-g dominant), C3 (inflammatory), C4 (lymphocyte depletion), C5 (immunologically quiet), and C6 (TGF-b dominant).

**Figure 6:**
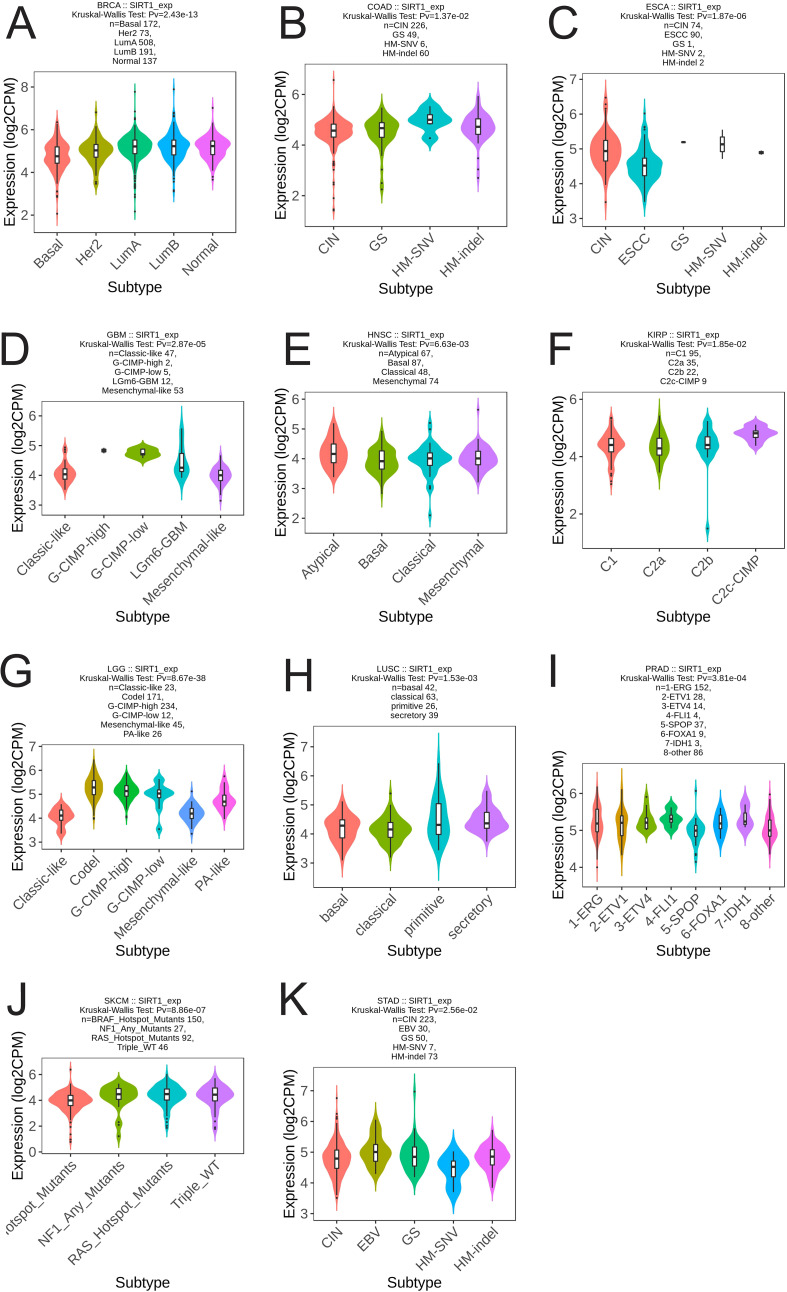
Correlation analysis between SIRT1 expression and molecular subtypes in 11 cancer types. **(A)** BRCA, **(B)** COAD, **(C)** ESCA, **(D)** GBM, **(E)** HNSC, **(F)** KIRP, **(G)** LGG, **(H)** LUSC, **(I)** PRAD, **(J)** SKCM, **(K)** STAD.

We analyzed the differential expression of SIRT1 in different immune and molecular subtypes in pan-cancer. The results revealed that SIRT1 was significantly different in different immune subtypes of 16 cancer types, including BLCA (5 subtypes), BRCA (5 subtypes), GBM (3 subtypes), HNSC (5 subtypes), KIRC (6 subtypes), LGG (4 subtypes), LIHC (5 subtypes), LUAD (5 subtypes), LUSC (5 subtypes), OV (4 types) subtypes), PAAD (5 subtypes), PRAD (4 subtypes), SARC (5 subtypes), SKCM (5 subtypes), TGCT (4 subtypes), and UCEC (5 subtypes) (**Figures 5A-P**). Additionally, significant changes in SIRT1 expression were observed in 11 different cancers based on molecular subtypes, including BRCA, COAD, ESCA, GBM, HNSC, KIRP, LGG, LUSC, PRAD, SKCM, and STAD (**Figures 6A-K**).

### Variation analysis of SIRT1 in pan-cancer

**Figure 7:**
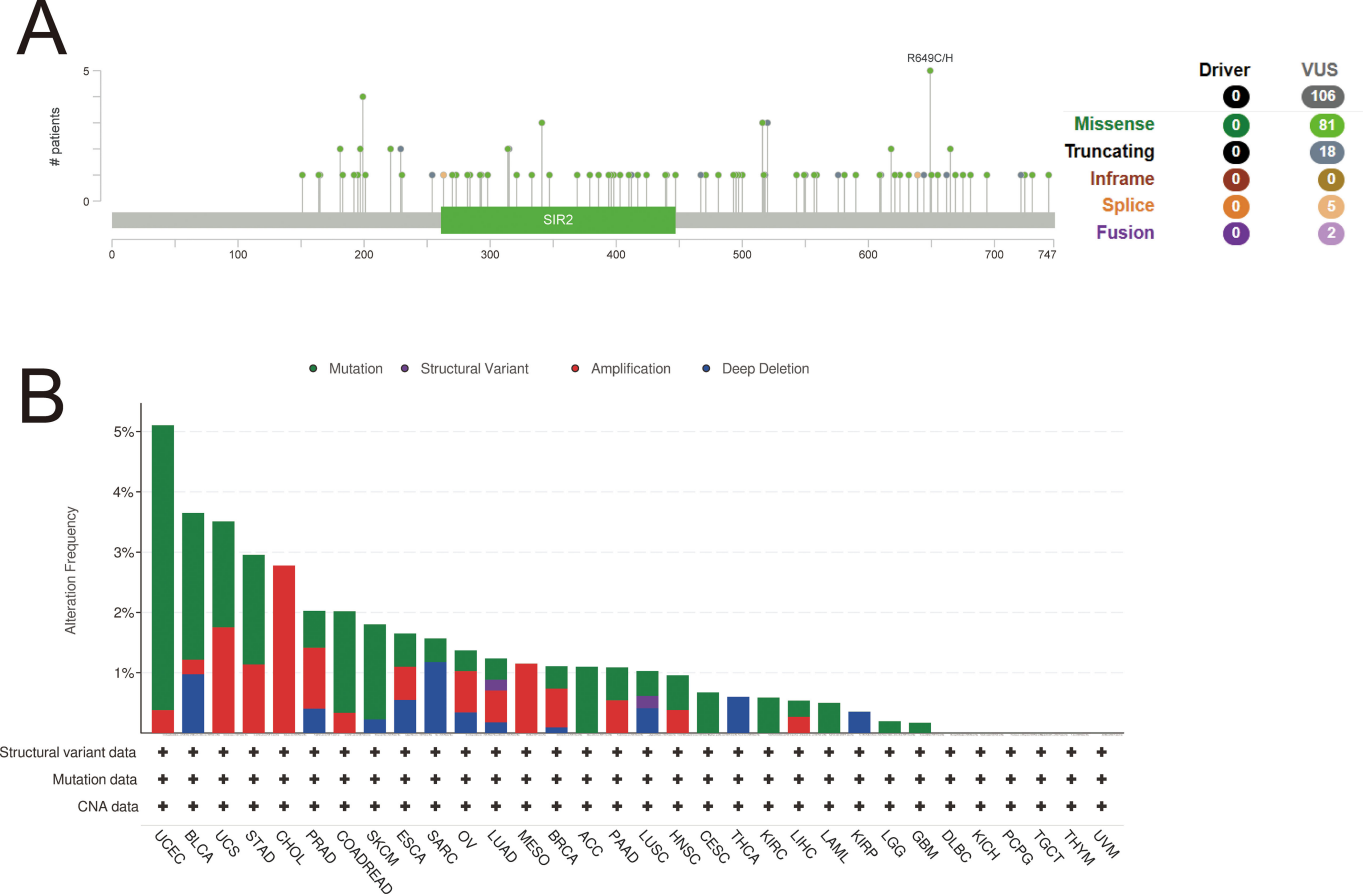
Variation analysis of SIRT 1 in 32 cancers. **(A)** Mutation map of SIRT1 across protein domains. **(B)** Bar chart of SIRT1 mutations in 32 cancers based on the TCGA pan-cancer atlas study.

Using the cBioPortal online website, gene mutations of SIRT1 in pan-cancer were analyzed. A total of 106 mutation sites were identified, spanning amino acids 0 to 747. These mutations consisted of 81 missense mutations, 18 truncation mutations, five splicing mutations, and two fusion mutations, with the most prevalent mutation being R649C/H (**Figure 7A**). The primary mutation types observed were missense mutations, amplifications, and deep deletions. SIRT1 mutations were most frequent in UCEC, BLCA, UCS, STAD, and CHOL (**Figure 7B**).

### PPI and functional enrichment analysis of SIRT1

**Figure 8:**
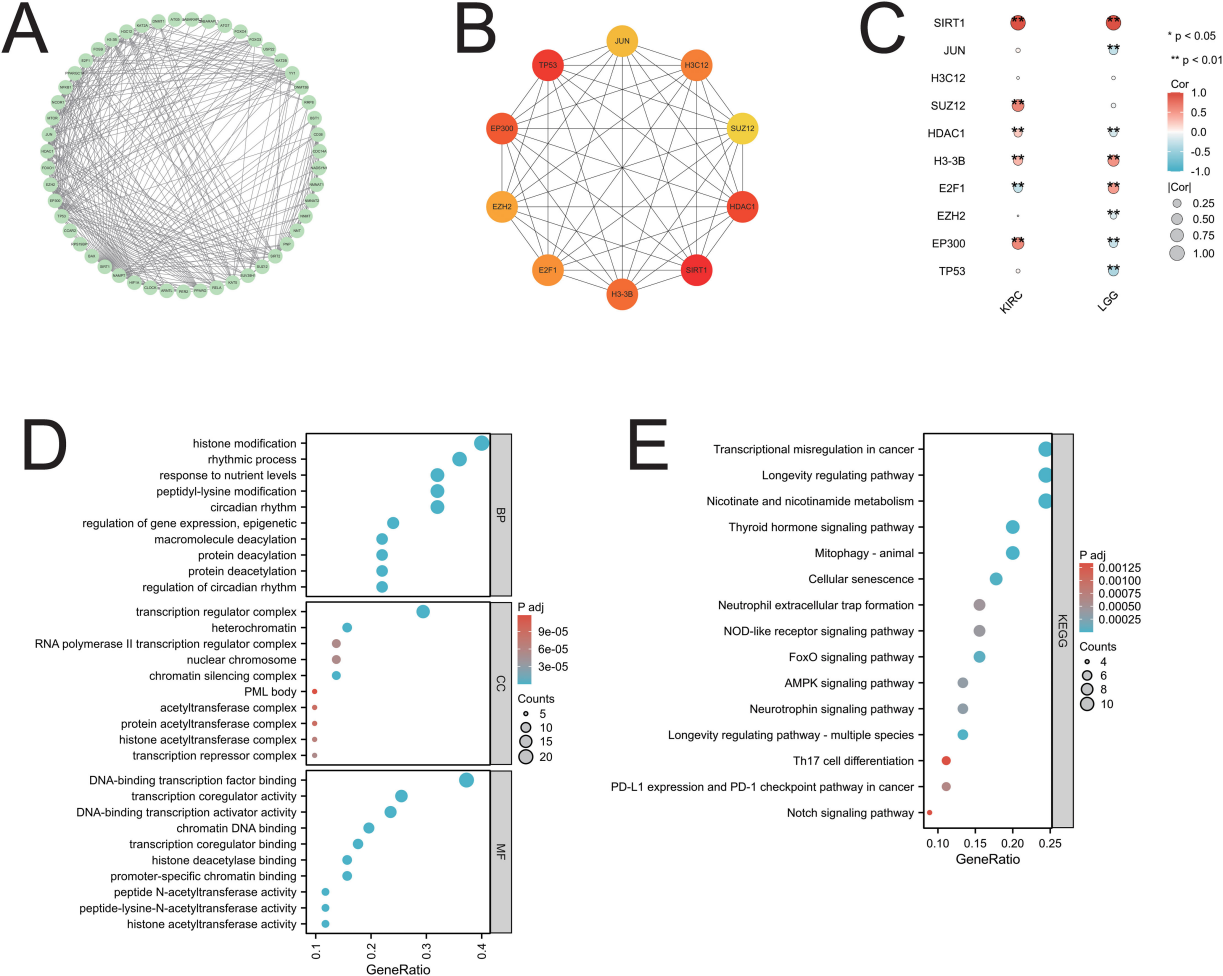
PPI network and functional enrichment analysis of SIRT1. **(A)** The PPI network of SIRT1; **(B)** the top ten core genes of the PPI network; **(C)** the core genes associated with SIRT1 in 2 cancers are presented in the form of a heatmap; **(D)** the GO enrichment analysis of SIRT1; **(E)** the KEGG pathway enrichment analysis of SIRT1. *p < 0.05, **p < 0.01.

Fifty genes closely related to SIRT1 were obtained from the STRING database and used to construct a PPI network on the basis of specific thresholds (**Figure 8A**). Analysis of the PPI network revealed ten core genes: JUN, H3C12, SUZ12, HDAC1, SIRT1, H3-3B, E2F1, EZH2, EP300, and TP53 (**Figure 8B**). Among these core genes, all except H3C12 were closely related to cancers (KIRC and LGG), in which SIRT1 expression affected prognosis (**Figure 8C**). GO enrichment analysis revealed that in the biological process (BP) category, the main GO terms included histone modification, peptidyl-lysine modification, macromolecule deacetylation, and protein deacetylation. In the CC category, the main GO terms included transcription regulator complex, heterochromatin, RNA polymerase II transcription regulator complex, and nuclear chromosome, among others. The main GO terms associated with molecular function (MF) were DNA-binding transcription factor binding, transcription coregulator activity, DNA-binding transcription activator activity, and chromatin DNA binding (**Figure 8D**). Furthermore, KEGG enrichment analysis revealed that these genes were involved in pathways such as transcriptional misregulation in cancer, mitophagy-animal, cellular senescence, and neutrophil extracellular trap formation (**Figure 8E**). The GO and KEGG pathway enrichment analysis results of SIRT1 can be found in **Supplementary Table S3**.

### GSEA analysis of SIRT1

**Figure 9:**
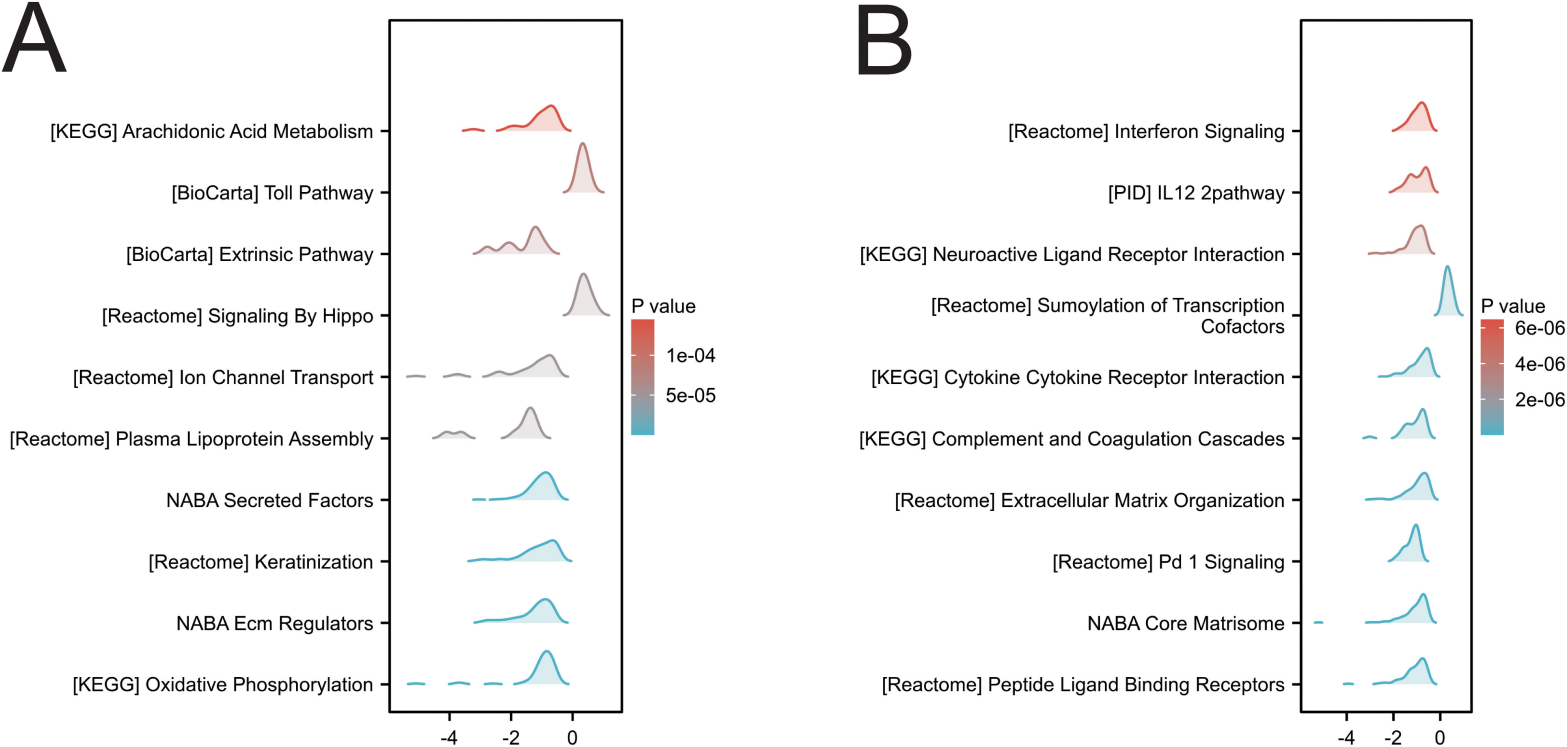
GSEA functional enrichment analysis of SIRT1 expression in 2 cancers. **(A)** KIRC, **(B)** LGG.

In two cancers (KIRC and LGG) associated with SIRT1 prognosis, GSEA analysis revealed multiple significantly enriched biological pathways (**Figures 9A, B**), including arachidonic acid metabolism, the Toll pathway, signaling by Hippo, keratinocyte, oxidative phosphorylation, interferon signaling, the IL12 pathway, neuroactive ligand–receptor interaction, cytokine–cytokine receptor interaction, extracellular matrix organization, the NABA core matrisome and peptide ligand-binding receptors. These results indicate that SIRT1 activity is closely related to DNA replication, repair, recombination, and transcriptional regulation in KIRC and LGG, reflecting its multifaceted role in tumor development and prognosis.

### Functional role of SIRT1 in cancer across single-cell states

**Figure 10:**
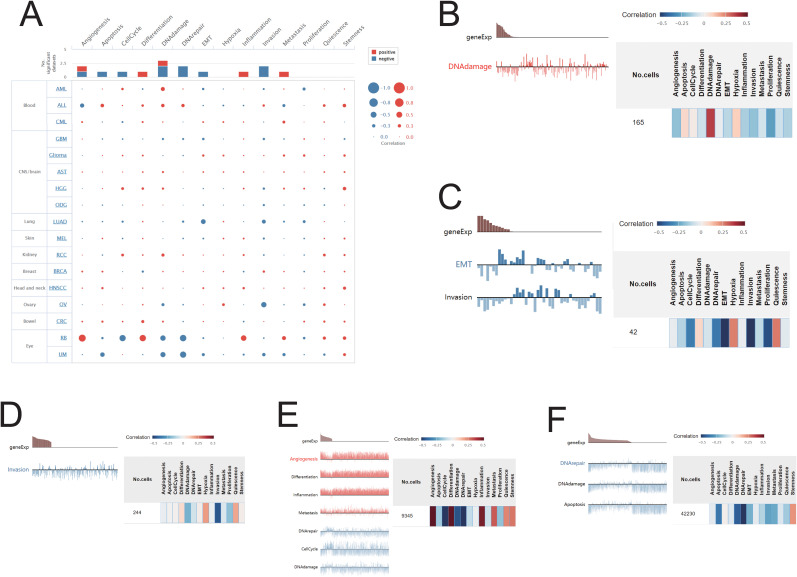
Correlations between SIRT1 and cancer functional status. **(A)** Bubble plot showing the correlation of SIRT1 with functional status in 17 cancer types. The correlation of SIRT1 with the functional state in **(B)** AML, **(C)** LUAD, **(D)** OV, **(E)** UM, and **(F)** RB.

Using the CancerSEA platform, we investigated the functional implications of SIRT1 across various types of cancer, revealing a significant correlation between SIRT1 expression and key cellular functions at the single-cell level. Our findings revealed positive associations between SIRT1 and processes such as DNA repair, DNA damage, stemness, and apoptosis but negative correlations with angiogenesis and metastasis (**Figure 10A**). Further investigation into specific cancer types revealed distinct functional relationships with SIRT1: in AML, SIRT1 was positively correlated with DNA damage; in LUAD, it was negatively correlated with EMT and invasion; in OV, it was negatively correlated with infection; and in UM, it was negatively correlated with apoptosis, DNA repair, and DNA damage. Notably, in RB, SIRT1 was positively correlated with angiogenesis, inflammation, differentiation, and metastasis but negatively correlated with DNA repair, the cell cycle, and DNA damage (**Figures 10B–F**).

### Immunogenomic analyses of SIRT1 in the pan-cancers

**Figure 11:**
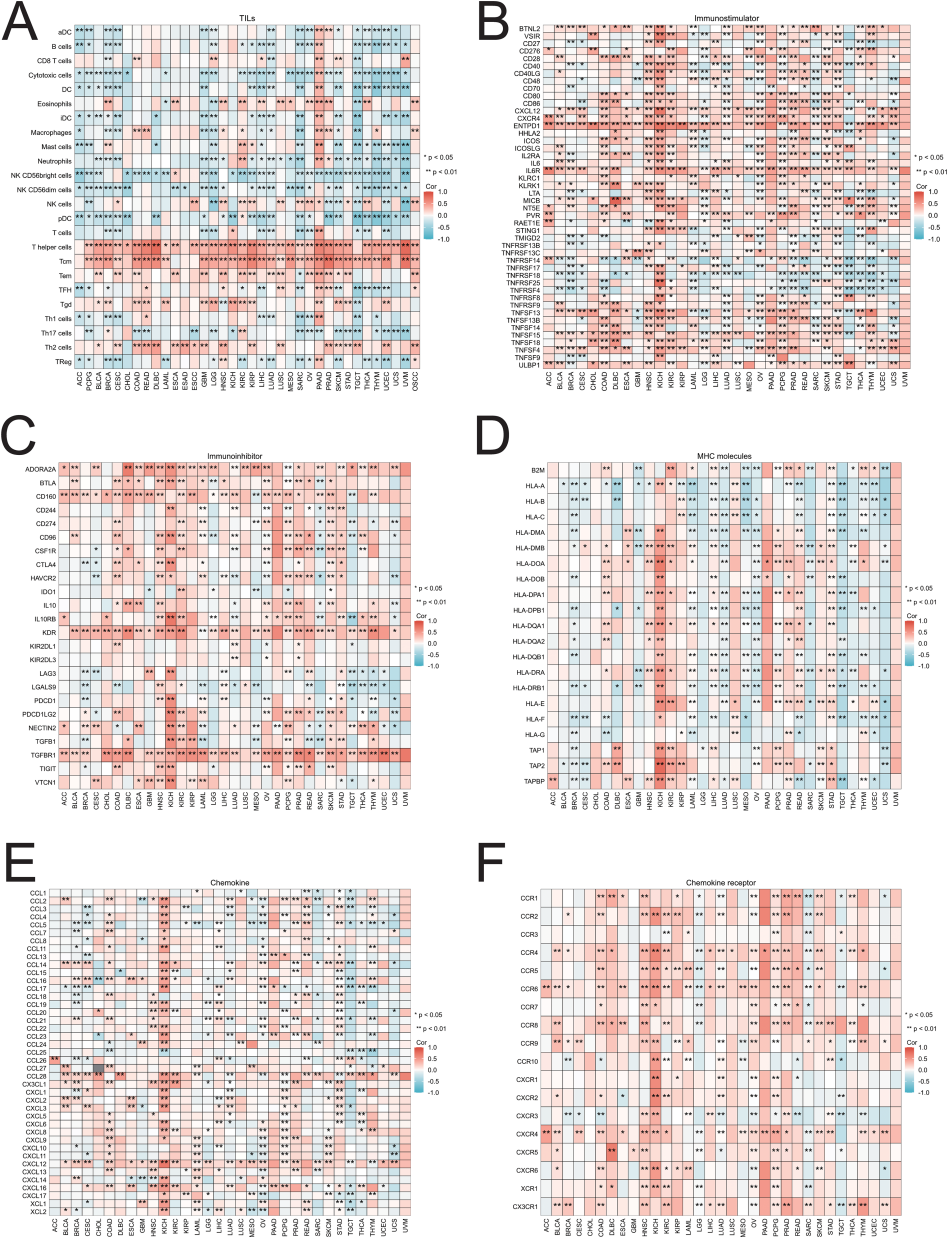
Correlations between SIRT1 and TILs and immune regulation-related genes in 33 cancer types. Correlations between the expression of SIRT1 and **(A)** TILs, **(B)** immunostimulators, **(C)** immunoinhibitors, **(D)** MHC molecules, **(E)** chemokines, and **(F)** chemokine receptors. *p < 0.05, **p < 0.01.

To investigate the relationships between SIRT1 and immune infiltration and regulation, we analyzed the correlations between SIRT1 and TILs, as well as immune cytokine markers, across various cancer types. The findings revealed that in 33 cancers, SIRT1 was positively correlated with the levels of eosinophils, T helper cells, central memory T cells (Tcm), effector memory T cells (Tem), γδ T cells (Tgd), and Th2 cell infiltration and negatively correlated with most other immune cell types (**Figure 11A**). In most cases, SIRT1 was positively correlated with immunostimulators, especially in HNSC, KICH, OV, PCPG, PRAD, and SKCM (**Figure 11B**). Additionally, SIRT1 was positively correlated with most immunoinhibitors, especially in COAD, HNSC, OV, PCPG, PRAD, and SKCM (**Figure 11C**). The analysis also revealed a positive correlation between SIRT1 and most MHCs in HNSC, KICH, KRC, LIHC, PRAD, and STAD, whereas it was negatively correlated with most MHCs in BRCA, CESC, LUAD, MESO, SARC, TGCT, and UCS (**Figure 11D**). Most chemokines in COAD, HNSC, KIRC, LIHC, PRAD, READ, and STAD were positively correlated with SIRT1, whereas in BRCA, UCS, SARC, and TGCT, they were negatively correlated (**Figure 11E**). In terms of chemokine receptors, SIRT1 was positively correlated with most cytokine receptors in COAD, HNSC, KIRC, and PRAD, whereas it was negatively correlated with LGG, SARC, and TGCT (**Figure 11F**).

### Molecular validation of SIRT1 in KIRC

**Figure 12:**
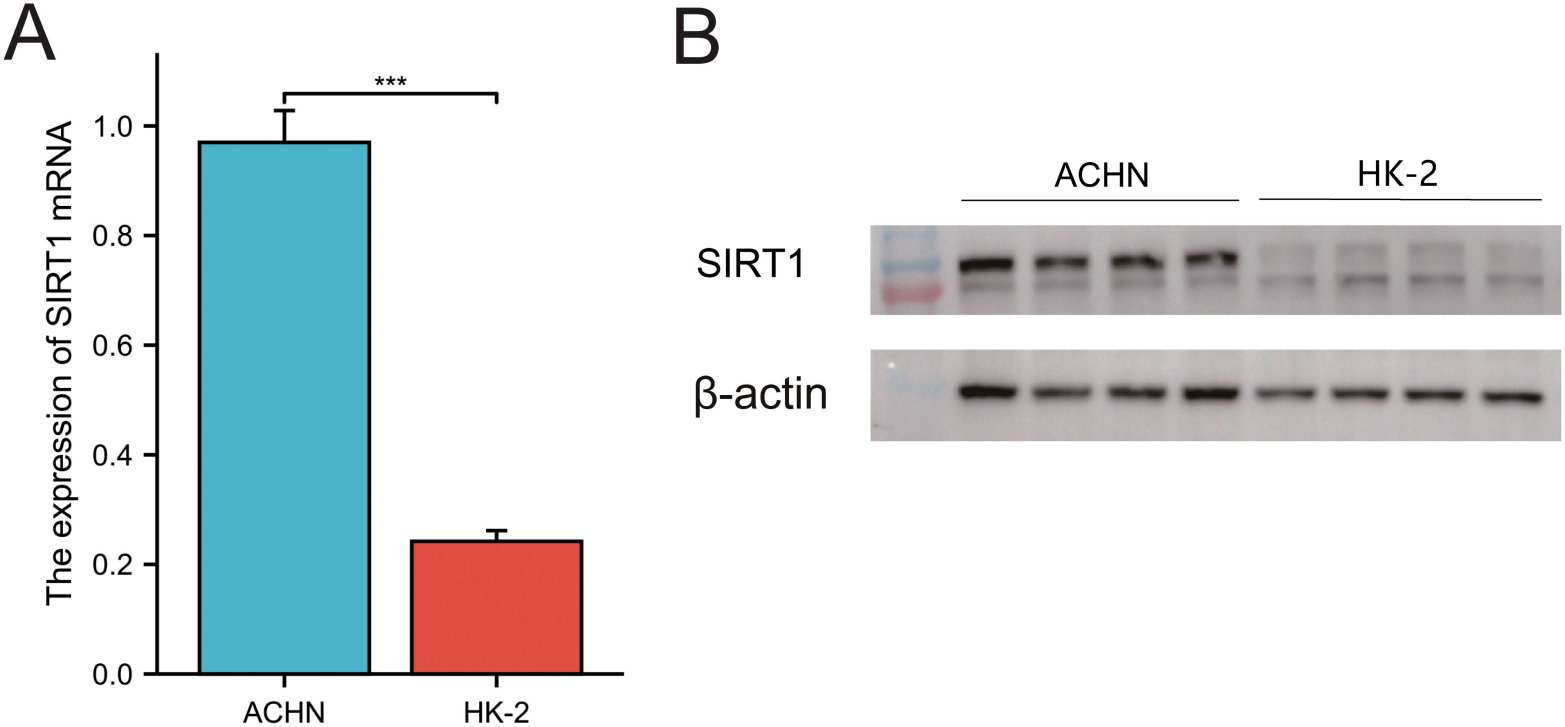
Expression of SIRT1 in KIRC. **(A)** qRT–PCR was used to analyze the expression of SIRT1. **(B)** WB was used to detect the relative expression of SIRT1. ***p < 0.01.

The results of qRT–PCR and Western blotting demonstrated that SIRT1 mRNA and protein levels were significantly greater in ACHN cells than in HK-2 renal tubular epithelial cells (**Figures 12A, B**). This observation is consistent with our analysis based on the TCGA database.

## Discussion

SIRT1 is an NAD+-dependent deacetylase involved in important processes such as cellular metabolism, DNA repair, and the stress response. Owing to its key role in regulating cellular function, energy balance, and aging, SIRT1 has attracted much attention in a variety of diseases, especially cancer. Through bioinformatics analysis, we comprehensively evaluated the function of SIRT1 in pan-cancer. First, we analyzed the mRNA and protein expression of SIRT1 in multiple human organs, tissues, and cell lines and compared its expression patterns in various cancers. We then evaluated the diagnostic and prognostic value of SIRT1 and explored its differential expression in different immune subtypes and tumor cell subtypes. Additionally, we identified the common mutation types and sites of SIRT1, constructed its PPI network, and analyzed its role in key cancer pathways. Finally, we validated the overexpression of SIRT1 in KIRC via qRT–PCR and Western blotting, confirming its potential as a therapeutic target. To our knowledge, this study is the first to comprehensively investigate the expression and biological function of SIRT1 from a pan-cancer perspective.

Through analysis of the HPA database, we found that SIRT1 is expressed at both the mRNA and protein levels in various organs and tissues, with notably higher expression in the adrenal gland, testes, ovaries, bone marrow, and lymph nodes. These results are consistent with those of previous studies, indicating that SIRT1 has a broad biological functional distribution in the immune system, endocrine system, and certain hematopoietic tissues (42). However, we observed significant differences in SIRT1 expression across different cancer types. For example, SIRT1 expression was significantly reduced in cancers such as ACC, BLCA, BRCA, CESC, and COAD (p < 0.05), whereas higher expression levels were observed in KIRC, LUAD, and STAD. These findings align with those of prior studies, suggesting that SIRT1 may play different biological roles in different tumor types (43, 44). In terms of diagnostic value, our study demonstrated that SIRT1 has high diagnostic sensitivity in various cancers, particularly in KICH and ESCC, where the AUC values were 0.923 and 0.902, respectively, indicating extremely high diagnostic efficacy. Other cancer types, such as BLCA, BRCA, LUSC, and PAAD, also exhibited strong diagnostic performance (AUC > 0.7). These results are consistent with the literature, further supporting the potential of SIRT1 as a diagnostic biomarker for cancer. For example, Desy et al. (4) reported that SIRT1 has excellent diagnostic efficacy in breast cancer, with an AUC value of 0.933, indicating that it has high diagnostic sensitivity (45). Therefore, SIRT1 not only serves as a potential therapeutic target but also as a valuable biomarker for early cancer diagnosis.

Our study used KM analysis to evaluate the prognostic value of SIRT1 in 33 types of cancer. In KIRC and LGG, patients with high SIRT1 expression had significantly better OS than those with low SIRT1 expression. These results suggest that SIRT1 may play a protective role in these two cancers. This finding is consistent with those of previous studies. Tian et al. reported that the expression level of SIRT1 in KIRC tissue was significantly greater than that in normal tissue and was associated with a good patient prognosis (15). Another study showed that SIRT1 can inhibit tumor progression by inhibiting the activity of HIF-1α and reducing the ability of KIRC cells to adapt to hypoxic environments (46). In gliomas, studies have shown that SIRT1 activators can induce tumor cell apoptosis, suggesting that SIRT1 may be a potential target for glioma treatment (47). Notably, however, SIRT1 had the opposite effect on STAD compared with KIRC and LGG. STAD patients with high SIRT1 expression had shorter DSS. These results suggest that SIRT1 may play different roles in different cancers. Studies have shown that SIRT1 can accelerate the progression of gastric cancer by deacetylating β-catenin and promoting the activation of the Wnt signaling pathway (48). Therefore, the specific mechanism by which SIRT1 affects the occurrence and development of gastric cancer still needs to be further explored. Future studies should focus on the interaction between SIRT1 and the Wnt signaling pathway, as well as its role in different tumor microenvironments. In addition, we also found that the expression level of SIRT1 was significantly correlated with the PFI of patients with KIRC, LGG, and GBM and that it had a protective effect on all three cancers. The results of a recent meta-analysis revealed that high expression of SIRT1 was an independent prognostic factor for prolonged overall survival in patients with malignant tumors (14). This finding is consistent with the results of this study, further suggesting that SIRT1 may be a potential biomarker for determining tumor prognosis. In summary, these findings reveal the important role of SIRT1 in the prognosis of multiple cancers, but its function in different cancers may differ.

SIRT1 expression is significantly different in different cancer molecular and immune subtypes. Specifically, our analysis revealed that SIRT1 was differentially expressed in immune subtypes of 16 cancer types, including BLCA, BRCA, GBM, HNSC, and KIRC. This difference in expression pattern suggests that SIRT1 may participate in tumor immune regulation through multiple mechanisms. For example, in the C1 (wound healing) immune subtype, the high expression of SIRT1 may be related to its ability to promote angiogenesis and tissue repair. Recent studies have confirmed that SIRT1 can promote angiogenesis via the deacetylation of HIF-1α and the regulation of VEGF expression, providing support for tumor growth and metastasis (49). In the C2 (IFN-γ dominant) subtype, SIRT1 may play a role by negatively regulating the IFN-γ-mediated inflammatory response. Studies have shown that mice lacking SIRT1 exhibit a stronger IFN-γ response and antitumor immune response (50). For the C3 (inflammatory) subtype, changes in SIRT1 expression may affect the production of inflammatory factors and the infiltration of immune cells. Mechanistic studies have shown that SIRT1 can inhibit the release of inflammatory mediators by deacetylating key transcription factors, such as the NLRP3 inflammasome and NF-κB (51). In the C4 (lymphocyte-depleted) subtype, changes in SIRT1 expression may be closely related to the regulation of T-cell function. Recent studies have shown that SIRT1 can affect the activation and effector function of T cells by epigenetically modifying immune checkpoint molecules such as PD-1 (52). In addition, the mechanism of action of SIRT1 in the C5 (immune-silent) and C6 (TGF-β-dominant) immune subtypes has attracted much attention. Some studies suggest that SIRT1 may participate in maintaining the immunosuppressive tumor microenvironment by regulating the differentiation and function of MDSCs (53). The interaction between SIRT1 and the TGF-β signaling pathway may affect the EMT process and tumor immune escape (54). In addition to its expression in immune subtypes, our analysis revealed that SIRT1 was significantly differentially expressed in molecular subtypes of multiple cancers, such as BRCA, COAD, and ESCA. These findings suggest that SIRT1 may be involved in regulating the activity of specific cancer-driving gene mutations or signaling pathways.

We analyzed mutations in the SIRT1 gene across cancers via the cBioPortal online database and found that SIRT1 mutations were most frequent in UCEC, BLCA, UCS, STAD, and CHOL. These findings differ from those of previous studies. For example, earlier research reported that SIRT1 mutations were more common in colorectal cancer and non-small cell lung cancer (55, 56). Several factors could contribute to these discrepancies. First, the sample sizes and types included in different studies may vary, leading to biased results. Second, with the advancement of sequencing technologies and the increase in sample sizes, new mutation patterns can be identified in larger datasets. Despite the widespread occurrence of SIRT1 mutations across various cancers, the mechanisms underlying its role in cancer initiation and progression may differ. In UCEC, SIRT1 mutations may promote tumor cell proliferation and invasion by affecting estrogen signaling pathways (57). In contrast, in STAD, SIRT1 mutations may be associated with Helicobacter pylori infection and inflammatory responses (58). These findings suggest that the biological function of SIRT1 mutations should be studied with consideration of the specificity of different cancer types. In particular, in different tumor microenvironments, SIRT1 mutations may exert their effects through distinct signaling pathways or molecular mechanisms. Furthermore, SIRT1 mutations may be correlated with the clinical features and prognosis of cancer. For example, in PAAD, SIRT1 mutations are negatively correlated with overall survival (59), whereas in BRCA, SIRT1 mutations are associated with increased tumor grade and lymph node metastasis (54). These results suggest that SIRT1 mutations may serve as potential prognostic biomarkers to guide the clinical management of cancer. However, this hypothesis still needs validation in larger clinical cohorts. Recent studies have indicated that SIRT1 mutations may lead to altered enzymatic activity, thereby affecting downstream gene expression and signaling pathways and playing a key role in tumorigenesis and progression (60). However, different types of SIRT1 mutations may have different impacts on its function, necessitating specific analysis of the relationship between mutation types and tumors. In summary, further investigations into the functional consequences of SIRT1 mutations will help elucidate their specific roles in the mechanisms of tumorigenesis.

Functional enrichment analysis of SIRT1 revealed that SIRT1 regulates cancer development by activating or inhibiting several known key pathways in cancer. These results suggest that SIRT1 may be involved in the occurrence and progression of tumors through multiple mechanisms. Wang et al. reported that inactivation of SIRT1 and AMPK in the SIRT1/AMPK pathway was associated with increased proliferation, migration, and invasion in renal cell carcinoma. The activation of SIRT1 can inhibit the malignant behavior of renal cell carcinoma cells by restoring the activity of AMPK and inducing cell apoptosis, thereby weakening the invasiveness and migration ability of tumor cells (61). In the SIRT1/p53 pathway, SIRT1 hinders p53 activity by deacetylating it and preventing apoptosis. Wang et al. demonstrated that SIRT1 inhibits the activity of p53 via deacetylation; downregulates the expression of miR-101; and increases the level of KPNA3, thereby promoting the proliferation, migration and invasion of colorectal cancer and enhancing the resistance of tumors to the chemotherapy drug 5-FU (62). Dasgupta et al. reported that the upregulation of SIRT1 in the SIRT1/NOX4 pathway activated the NOX4-mediated oxidative stress response, leading to muscle breakdown and adipose tissue consumption, thereby exacerbating the occurrence of cancer cachexia. Inhibition of the SIRT1-NOX4 signaling axis can significantly alleviate the symptoms of pancreatic cancer-related cachexia (63). Moreover, in breast cancer cells, SIRT1 and FOXO4 collaborate to prevent cell apoptosis and promote tumor cell survival (64).

Our analysis of the correlation between SIRT1 and immune cell infiltration in 33 cancer types yielded results that were consistent with those of previous studies. We found that SIRT1 was positively correlated with the infiltration of eosinophils, T helper cells, Tcm, Tem, Tgd, and Th2 cells in most tumors but negatively correlated with the majority of other immune cell types. These findings align with the known role of SIRT1 in regulating immune cell infiltration, particularly that of macrophages and T cells, as reported in earlier studies (65). In the context of cutaneous T-cell lymphoma (CTCL), our observation that SIRT1 inhibition induces cell growth arrest and apoptosis corroborates previous work highlighting its potential role in controlling immune cell proliferation and infiltration. These findings are further supported by studies demonstrating the involvement of SIRT1 in macrophage polarization toward the antitumor M1 phenotype via the NF-κB pathway and its ability to modulate B-cell activation through the PI3K/Akt/eNOS signaling axis (65, 66). Our findings on the impact of SIRT1 on Th2 immune responses through the regulation of transcription factors and metabolic pathways are consistent with prior research. For example, the inhibition of IL-9 production and subsequent prevention of Th9 cell differentiation by SIRT1 via histone deacetylation, as observed in our study, has been previously reported (67). Moreover, our results support the crucial role of mTOR-HIF1α axis-mediated glycolytic pathway activation in this differentiation process, confirming the dual role of SIRT1 in metabolic and transcriptional regulation, as suggested by earlier studies (60). The role of SIRT1 in maintaining memory T cells, particularly Tcm T cells, which we found to be positively correlated with SIRT1 expression, was previously attributed to its regulation of the PI3K/Akt/eNOS axis (66). Our observations of increased effector memory T-cell responses and reduced initial T-cell numbers under SIRT1-deficient conditions further emphasize its importance in immune memory formation, which is consistent with prior reports (68). With respect to γδ T cells, our findings on the regulatory function of SIRT1 through its influence on NF-κB, which enhances metabolic signaling for swift response capability, are in line with previous studies (69). The ability of SIRT1 to control inflammatory pathways and support glycolytic metabolism, as observed in our analysis, has been previously shown to enhance the function and proliferation of γδ T cells. In summary, our results confirming the ability of SIRT1 to regulate immune cell infiltration, Th2 immune responses, memory T-cell persistence, and γδ T-cell function are consistent with previous studies, reinforcing its key role in immune cell biology. The regulatory mechanisms involving transcriptional and metabolic pathways identified in our work have been supported by prior research, providing further insights into the potential of targeting SIRT1 for immune modulation in cancer and autoimmune diseases.

Finally, via qRT–PCR and WB experiments, we validated the upregulation of SIRT1 expression in KIRC, confirming previous findings. In summary, this study provides compelling evidence supporting the oncogenic role of SIRT1 across multiple cancers and highlights its promising potential as a therapeutic target for cancer treatment.

However, there are several limitations to this study. First, the sample sizes for certain tumor types in the database are relatively small, and variations in sequencing methods across different platforms and databases (e.g., TCGA, GTEx) may impact the accuracy and consistency of the data. These methodological differences, including variations in sequencing technologies and data normalization techniques, could affect the reliability and generalizability of the findings. Second, this study presents only preliminary evidence of the associations between SIRT1 and various cancers. Further experiments are needed to explore the specific molecular functions and mechanisms of SIRT1 in tumorigenesis, which will require the integration of multiomics data and the application of advanced experimental techniques. Additionally, the high heterogeneity of cancer may influence the effectiveness and specificity of SIRT1 as a diagnostic or prognostic biomarker, and the therapeutic efficacy of SIRT1-based interventions may vary among individual patients. Addressing these challenges will be crucial for translating the current findings into clinical practice. Nevertheless, this study provides a robust foundation for understanding the role of SIRT1 in cancer and offers valuable insights for the future development of precision-targeted therapies and immunotherapies.

## Conclusions

This study systematically elucidates the role of SIRT1 in pan-cancer from multiple perspectives, including gene expression, prognosis, function, mutation sites, and immune cell infiltration. These findings reveal that SIRT1 expression is significantly downregulated in multiple cancers and displays differential expression across distinct molecular and immune subtypes. In most cancer types, increased SIRT1 expression is positively associated with eosinophils, helper T cells, central memory T cells, effector memory T cells, γδ T cells, and Th2 cells. Moreover, SIRT1 expression is strongly associated with immunoregulatory factors across different cancers. SIRT1 is capable of activating or inhibiting multiple cancer-related pathways and is intricately involved in immune infiltration and immune regulation. Our study provides a solid foundation for understanding the role of SIRT1 in cancer and provides valuable insights for the development of future precision targeted therapies and immunotherapies.

## Data Availability

The original contributions presented in the study are included in the article/**Supplementary Material**. Further inquiries can be directed to the corresponding authors.

## Supplementary material

Supplementary Figure 1 Detailed information of SIRT1 mRNA expression in tissues and cell lines. **(A)** Detailed information of SIRT1 mRNA expression in different tissues, **(B)** Detailed information of SIRT1 mRNA expression in different cell lines.
